# Progress in Dermatology

**Published:** 1900-07-14

**Authors:** 


### Progress in Dermatology.

**Lupus.**—Leredde,[1] in an exhaustive paper upon tuberculosis of the skin, urges that the following diseases are directly connected with the tubercle bacillus. They are lupus erythematosus, Bazin's disease, acinitis of follicles of Barthelemy, erythema indurata, lichen scrofulosorum, acne cachetica, eczema scrofulosorum, and the cachetic gangrene of children. Though none of these diseases yield tubercle bacilli, yet they have a marked affinity to tuberculosis, and it is suggested that they are caused by poisoning with the toxins of the bacillus.

Johnston[2] concludes that there is a class of cutaneous disease analogous to the para-syphilomata, which may be called para-tuberculous. They are not in themselves tuberculous, but they develop in tuberculous soil. They may be divided into three groups: Scrofulodermata, tuberculoderms, and dyschromias. The former are pustular, the tuberculoderms are toxæmias, and include a variety of affections, ranging from erysipelas perstans to lichen scrofulosorum. The diseases which should be placed in these categories are those in which there is an absence of tubercle bacilli; the pathological anatomy is comparable to that recognised for tuberculosis, and which can be experimentally produced by injections of tuberculin.

Mackenzie[3] describes a visit paid to the Finsen Lysinstitut. In the cured cases of lupus the scars were good, being supple and without contraction. The result of the application of the treatment to mucous membranes had also been successful. In some cases there is considerable inflammation excited, and occasionally true erysipelas. The treatment is not equally suitable for all cases, but it is preferable to erasion or escharotics; it should not, however, be employed where complete excision is feasible. The objections to the treatment are: (1) The length of time (*i.e.*, for three or four months), involving a daily attendance; (2) the expense, not only of the apparatus, but for the nursing, since a special nurse is required for each case.

Valdemar Bie[4] describes the method and apparatus adopted by Finsen, and insists on the bactericidal properties of the violet end of the spectrum and on the power of these rays to penetrate deeply into the cutis and to produce inflammation. This power is increased when the skin is rendered anæmic.

Thurston Holland[5] gives an account of two cases of tuberculous dermatitis treated by the *x* rays. In the first case the dorsum of the foot had been affected with tubercular ulceration for 11 years and had resisted all treatment; the second case was one of lupus of the head and neck in a boy of five years' duration. In two months 19 exposures were given to the first case and 17 to the second; both cases quickly improved, and were healed when seen some months afterwards; in each case, *x* ray dermatitis was produced, causing loss of the nails in the first and alopecia in the second case; both hair and nails quickly grew again.

Philippson[6] advises the internal administration of fluoride of sodium in 1 decigram doses for the treatment of lupus. He has given it in the form of pills and by hypodermic injections. He considers that the drug has a curative effect, but it has such a disturbing effect on the stomach that its administration cannot be continued until cure is complete; as an alternative the fluoro-benzoate of soda in 50 cg. doses may be given. No perfect cure has yet been obtained.

Little[7] showed a case of lupus vulgaris affecting the scars left after vaccination. The vaccination itself was quite normal, but within six weeks lupoid nodules appeared in one of the scars. It is therefore supposed that the disease must have been inoculated with the vaccine.

**Acne.**—Levieur[8] recommends the use of iodide of potassium in obstinate cases of acne vulgaris. This drug produces increase of inflammation in the eruption. When sufficient inflammation has been obtained its use should be stopped and some local remedies substituted. It is given in 5 grain doses in milk, and the urine is tested frequently to see whether iodine is present; when it is found, the iodide should be stopped and mild sulphur ointment substituted.

Sands[9] states that acne includes 8 per cent. of all skin diseases, that it includes no less than 17 distinct diseases. The typical disease commences as a superficial hyperkeratosis, and becomes a hyperidrosis oleosa; it then becomes inflammatory, with hypertrophy of the sebaceous glands, and the formation of inflammatory nodes (acne indurata) or of pustules (acne pustulata). He did not agree with the dependance of the eruption upon general systemic disturbance, since he found that 70 per cent. of his acne cases were free from constipation. He had never seen a case recover under sulphur ointment, so he recommends an ointment containing zinc oxide, 10; resorcin, 40; and benzoated lard, 50 parts. In the case of pustular acne, he inserts a drop of pure carbolic acid into the centre of each pustule, followed by a salicylic plaster.

Whitfield[10] relates a case of acne vulgaris combined with seborrhoic eczema; this combination was peculiar, since seborrhoic eczema is usually considered to be related to psoriasis. He was treating it with an ointment containing 2 per cent. of salicylic acid and 3 per cent. of sulphur; if the disease did not yield to this he should add some oil of cade.

Sabouraud[11] arrives at the following conclusions about necrotic acne: (1) Necrotic acne is a perifolliculitis of the superficial and deeper parts of the skin, terminating in necrosis of all tissues invaded, and in a depressed variola-like cicatrix. (2) The lesion commences in a follicle previously infected by the micro-organism of fatty seborrhœa. (3) This affection is produced by a yellow staphylococcus, which is constantly and abundantly found. (4) This cannot be distinguished from the ordinary staphylococcus aureus. This fact, taken in connection with the clinical characters of necrotic acne, tend to bring into the same group (*a*) the peripapillary impetigo of Bockhart; (*b*) impetigo pilaris of the adult; and (c) true necrotic acne. The treatment of an acute attack is simple, but it is very difficult to prevent recurrences. In fact, the micro-bacillary fatty seborrhœa, which is the constant substratum, must first be cured.

Touton[12] defines acne diseases as those conditions in which the primary lesion is an inflammation of the hair follicles, with a tendency to suppuration, chiefly involving the sebaceous glands, and in which the individual lesions run an acute course. He discusses the various forms of acne which do not, in his opinion, belong to this class, and concludes by forming a group of (1) acne vulgaris, (2) tar and other acnes of external origin, and (3) iodide and bromide acnes, and others of internal origin.

Muller[13] recommends the treatment of acne by steam baths, combined with the use of a soap containing some combination of resorcin, salicylic acid, sulphur, and balsam of Peru. He lathers the soap well into the skin, and partly removes it with luke-warm water, allowing the remainder to dry into the skin.

**Pemphigus.**—Marx[14] records a series of cases of contagious pemphigus starting in a three-day-old infant. It commenced as a vesicle on the head, and from thence it spread all over the body and extremities; finally the child died on the ninth day. On the sixth day of the child's disease the mother developed bullæ, first on the mamma, then in the groins and over the body; later the nurse had a vesicle on the neck, and at the same time the father and his three sons all developed some vesicles; lastly, the physician got a vesicle on the forearm.

Kohler[15] describes some cases of acute contagious pemphigus affecting adults. Five persons (four children and one adult), who lived in two neighbouring houses, were attacked with the disease, and all recovered except one infant, who died in the hospital. A week after the sick child's admission the nurse was affected with blebs, the first of which appeared on the face; two days later another nurse, who had washed the dead child's linen, was also affected, the blebs appearing first on the arms. In all, seven persons were affected, three of whom were adults. Staphylococci and diplococci were found in the blebs.

Leredde[16] considers that pemphigus foliaceous is a blood disease, revealed clinically by the phenomena which it provokes. He found microscopically that there was a marked diapedesis of eosinophyllic cells into the dermis, and an increase of eosinophil cells in the blood. The lesions of the skin and of the blood resembled those found in Duhring's disease, and he thinks that pemphigus vegetans, as well as Duhring's disease, depend upon an affection of the bone marrow set up by various toxins. In fact, he thinks that the blood serum is charged with substances which provoke cutaneous irritation, and that these substances are produced in the bone marrow.

Lalanne[17] publishes an interesting case of pemphigus in a patient suffering from general paralysis. Following the outbreak, an attack of infective dermatitis was communicated to the nurse; the disease was supposed to be trophic in origin, although there was history of previous syphilis. The reason for not considering the eruption specific was that syphilitic pemphigus is confined to young infants.

[1] La Sem. Med., Jan. 3, 1900, p. 1. [2] Jour. of Cut. and Genito-Urinary Dis., July, 1899. [3] Brit. Jour. of Derm., Nov., 1899, p. 427; Clin. Jour., Nov., 1899, p. 49. [4] Brit. Med. Jour., Sept. 30, 1899, p. 825; Med. Chron., Dec., 1899, p. 208. [5] Brit. Med. Jour. Ep., Nov. 4, 1899, No. 304; Arch. of Röntgen Rays, May 1899. [6] Brit. Med. Jour. Ep., Nov. 26, 1899, No. 418; Derm. Zeitch, 1899, p. 289. [7] Brit. Jour. of Derm., Feb., 1900, p. 58. [8] Med. Rec., Nov. 11, 1899, p. 700. [9] Ditto, Jan. 20, 1900, p. 123. [10] Brit. Med. Jour., Feb. 10, 1900, p. 319; Lancet., Feb. 10, 1900, p. 385. [11] Ann. de Derm. et de Syph., Oct., 1899, p. 841; Med. Rec., Dec. 23, 1899, p. 929; Brit. Jour. of Derm, Feb., 1900, p. 71. [12] Ditto, Nov., 1899, p. 458. [13] Ditto, Jan., 1900, p 38; Derm. Zeitch, Vol 6, Nov., 1899, p. 583. [14] Med. Rec., St. Louis, Sept. 30, 1899; Int. Med. Mag., Nov., 1899, p. 835. [15] Deutch. Arch. fur Med., No. 62, p. 525; New York Med. Jour., Jan. 27, 1900, p. 131. [16] Ann. de Derm. et de Syph., July 19, 1899, p. 601; Brit. Jour. of Derm., Oct., 1899, p. 407. [17] Progrès Med., Sept. 23, 1899; Med. Rec., Oct. 21, 1899, p. 591.

